# The tubulin cofactor A is involved in hyphal growth, conidiation and cold sensitivity in *Fusarium asiaticum*

**DOI:** 10.1186/s12866-015-0374-z

**Published:** 2015-02-18

**Authors:** Xiaoping Zhang, Xiang Chen, Jinhua Jiang, Menghao Yu, Yanni Yin, Zhonghua Ma

**Affiliations:** Institute of Biotechnology, Zhejiang University, Hangzhou, China; Institute of Agriculture Quality and Standard for Agro-products, Zhejiang Academy of Agricultural Sciences, Hangzhou, China

**Keywords:** TBCA, Conidiation, Cold sensitivity, *F. asiaticum*, Hyphal growth, Fusarium head blight

## Abstract

**Background:**

Tubulin cofactor A (TBCA), one of the members of tubulin cofactors, is of great importance in microtubule functions through participating in the folding of α/β-tubulin heterodimers in S*accharomyces cerevisiae*. However, little is known about the roles of TBCA in filamentous fungi.

**Results:**

In this study, we characterized a TBCA orthologue FaTBCA in *Fusarium asiaticum*. The deletion of *FaTBCA* caused dramatically reduced mycelial growth and abnormal conidiation. The *FaTBCA* deletion mutant (ΔFaTBCA-3) showed increased sensitivity to low temperatures and even lost the ability of growth at 4°C. Microscopic observation found that hyphae of ΔFaTBCA-3 exhibited blebbing phenotypes after shifting from 25 to 4°C for 1- or 3-day incubation and approximately 72% enlarged nodes contained several nuclei after 3-day incubation at 4°C. However, hyphae of the wild type incubated at 4°C were phenotypically indistinguishable from those incubated at 25°C. These results indicate that *FaTBCA* is involved in cell division under cold stress (4°C) in *F. asiaticum*. Unexpectedly, ΔFaTBCA-3 did not exhibit increased sensitivity to the anti-microtubule drug carbendazim although quantitative real-time assays showed that the expression of *FaTBCA* was up-regulated after treatment with carbendazim. In addition, pathogenicity assays showed that ΔFaTBCA-3 exhibited decreased virulence on wheat head and on non-host tomato.

**Conclusion:**

Taken together, results of this study indicate that FaTBCA plays crucial roles in vegetative growth, conidiation, temperature sensitivity and virulence in *F. asiaticum*.

**Electronic supplementary material:**

The online version of this article (doi:10.1186/s12866-015-0374-z) contains supplementary material, which is available to authorized users.

## Background

Microtubules polymerized by α/β-tubulin heterodimers play a central role in many cellular processes, including cell divisions, intracellular transport processes, and cell polarity. Biosynthesis of tubulin heterodimers is a multistep process involving several molecular chaperones and tubulin cofactors (TBCs) [1]. In *Saccharomyces cerevisiae,* the nascent α- and β-tubulin polypeptides firstly interact with prefoldin and cytosolic chaperonin CCT (Cytosolic-Chaperonin-containing-TCP1) [2]. Subsequently, five TBCs (TBCA-E) participate in the tubulin folding pathway. TBCA and TBCB bind to β-tubulin and α-tubulin, respectively, which then transfer β-tubulin to TBCD and α-tubulin to TBCE respectively [3,4]. Afterwards, TBCC binds to the supercomplex containing TBCD, TBCE, α- and β-tubulin, and stimulates GTP hydrolysis resulting in the release of the α/β-tubulin heterodimers [5].

TBCA, as a β-tubulin-interacting protein, was first purified from bovine testis [6]. To date, characterization of several TBCA in yeast, murine, Arabidopsis and human has demonstrated that TBCA regulates both the ratio between α- and β-tubulin and the tubulin folding pathways for correct polymerization into microtubules [7-11]. Rbl2p, the TBCA yeast orthologue, is not an essential gene in *S. cerevisiae* but is required for normal meiosis [7]. In fission yeast *Schizosaccharomyces pombe*, TBCA orthologue Alp31 plays an important role in the maintenance of microtubule integrity and the determination of the cell polarity [11]. Mutations in *KIS* (*TBCA* orthologue) lead to defects similar to the phenotypes associated with impaired microtubule function in *Arabidopsis* [9]. TBCA knockdown by RNAi in human cell lines, results in decreased amounts of α- and β-tubulin levels, subtle alterations in the microtubule cytoskeleton, G1 cell cycle arrest and cell death [10].

*Fusarium asiaticum* (teleomorph: *Gibberella zeae*) is one of major causal agents that are responsible for economically important Fusarium head blight (FHB) on various cereal crops [12-15]. In addition to yield reduction, this pathogen produces mycotoxins including deoxynivalenol (DON), acetyldeoxynivalenol (3-ADON or 15-ADON), nivalenol (NIV), and acetylnivalenol (4-ANIV) in infected plants, which pose a serious threat to human and animal health [16,17]. In China, *F. asiaticum* is more important than *F. graminearum* with respect to population quantity and mycotoxin production [15]. Because highly resistant wheat cultivars are not available [18], chemical control remains one of the major strategies for the management of FHB [19]. However, highly effective fungicides against FHB are limited [19]. Moreover, *Fusarium* spp. have developed resistance to several commercialized fungicides [20-23]. Therefore, the exploration of new compounds and potential targets is desperately needed for an effective management of FHB. In the therapies of human diseases, TBCA has been regarded as an attractive target for the treatment of clear cell renal cell carcinoma (ccRCC), since it plays crucial roles in the progression, invasion and metastasis of ccRCC [24]. Until now, little is known about the roles of TBCA in filamentous fungi. In this study, we were thus interested in investigating the functions of TBCA in *F. asiaticum*, which may help in its exploitation as a drug target for the design of new antifungal agents against FHB.

## Methods

### Strains and culture conditions

*F. asiaticum* strain GJ33 collected from Jiangsu province, China was used as a wild-type strain in this study. The wild-type strain and the resulting transformants were grown on potato dextrose agar (PDA; 200 g potato, 20 g dextrose, 20 g agar and 1 l water), complete medium (CM; 1% glucose, 0.2% peptone, 0.1% yeast extract, 0.1% casamino acids, nitrate salts, trace elements, 0.01% vitamins and 1 l water, pH 6.5), minimal medium (MM; 10 mM K_2_HPO_4_, 10 mM KH_2_PO_4_, 4 mM (NH4)_2_SO_4_, 2.5 mM NaCl, 2 mM MgSO_4_, 0.45 mM CaCl_2_, 9 mM FeSO_4_, 10 mM glucose and 1 l water, pH 6.9), or wheat-head medium (200 g grounded fresh wheat heads and 20 g agar in 1 l water) for mycelial growth tests, and in carboxymethyl cellulose liquid medium (CMC; 15 g carboxymethyl cellulose, 1 g yeast extract, 0.5 g MgSO_4_, 1 g NH_4_NO_3,_ 1 g KH_2_PO_4_ and 1 l water), or mung bean agar (MBA; 40 g mung beans boiled in 1 l water for 20 min, and then filtered through cheesecloth, 20 g agar) for sporulation tests.

### Sequence analyse of *FaTBCA* gene from *F. asiaticum*

Based on the sequence of *TBCA* (FGSG_00510.3) gene in *F. graminearum*, one pair of primers A1 + A2 (Additional file 1) was designated to amplify *FaTBCA* from GJ33 DNA. PCR amplifications were purified, cloned, and sequenced. The sequence of *FaTBCA* was deposited in GenBank under accession number KM116518. On the basis of deduced amino acid sequences of FaTBCA and its orthologues, the phylogenetic tree was generated by the neighbor-joining method with 1000 bootstrap replicates using the Mega 4.1 software [25].

### Yeast complementation assays

Full-length cDNA of *FaTBCA* was amplified using primer pair A3 + A4 listed in Additional file 1. PCR product was digested with appropriate enzymes and cloned into the pYES2 vector (Invitrogen Co., CA, USA), and transformed into the corresponding yeast mutant. Yeast transformants were then selected on synthetic medium lacking uracil. Additionally, the wild-type strain BY4741 and the mutant transformed with an empty pYES2 vector were used as controls. For complementation assays, the yeast transformants were grown at 30°C on YPRG medium (1% yeast extract, 2% bactopeptone, 2% galactose) supplied with 0.48% (v/v) HCl. The experiments were repeated three times independently. There are four replicates for each experiment.

### Construction of gene deletion and complemented strains

*FaTBCA* deletion vector pBS-*FaTBCA*-Del was constructed by inserting two flanking sequences of *FaTBCA* into left and right sides of *HPH* (hygromycin resistance gene) in the pBS-HPH1 vector [26]. Briefly, by using primer pair A5 + A6 (Additional file 1), a 477 bp upstream flanking sequence of *FaTBCA* was amplified from GJ33 genomic DNA, and was inserted into *Xho*I-*Sal*I sites of the pBS-HPH1 vector to generate a plasmid pBS-*FaTBCA*-Up. Subsequently, a 417 bp downstream flanking sequence of *FaTBCA* amplified from GJ33 genomic DNA using the primers A7 + A8 (Additional file 1) was inserted into *Hind*III-*Bam*HI sites of the pBS-*FaTBCA*-Up vector to generate a plasmid pBS-*FaTBCA*-Del. Finally, the 2394 bp fragment containing *FaTBCA*-upstream-HPH-*FaTBCA*-downstream cassette was obtained by PCR amplification with primer pair A5 + A8 from the pBS-*FaTBCA*-Del. The resultant PCR product was purified and used for protoplast transformation. The PEG-mediated protoplast fungal transformation was performed as described previously [27]. For selective growth of transformants, PDA medium supplemented with hygromycin (100 mg l^−1^) were used.

To confirm that the phenotype of *FaTBCA* deletion mutant is due to disruption of the gene, genetic complementation was performed. The *FaTBCA* complement plasmid pCA-*FaTBCA*-Com was constructed using the backbone of pCAMBIA1300. First, a *Xho*I-*Kpn*I *neo* cassette containing a *trpC* promoter was amplified from plasmid pBS-RP-Red-A8-NEO [28] with primers Neo-F + Neo-R (Additional file 1), and cloned into the *Xho*I-*Kpn*I site of pCAMBIA1300 to create plasmid pCA-neo. Then, a 1862 bp of full length *FaTBCA* gene including 1437 bp promoter region was amplified using primer pair A11 + A12 (Additional file 1) from genomic DNA of the wild-type GJ33, and subsequently cloned into the *Kpn*I-*Xba*I sites of pCAMBIA1300 to generate the complement plasmid pCA-*FaTBCA-*Com. Transformation of ΔFaTBCA-3 with the full-length *FaTBCA* was conducted as described above except that geneticin (100 mg l^−1^) was used as a selection agent. After single spore isolation, all of the mutants generated in this study were preserved in 15% glycerol at −80°C.

### Microscopic examinations of hyphal and conidial morphology

Hyphal growth of each strain was tested on PDA, CM, MM and wheat-head media at 25°C for 3 days. The experiment was repeated three times, and each with two replicates. Hyphal morphology was examined under a Nikon ECLIPSE Ni-U microscope (Nikon Co., Tokyo, Japan) from mycelia that were incubated in potato dextrose broth (PDB; 200 g potato, 20 g dextrose and 1 l water) at 25°C for 1 day using a shaker then transferred to static incubation at 4, 10 and 25°C for 1 day or 3 days. Furthermore, nucleus of hyphae were examined under the same microscope after staining with 4',6-diamidino-2-phenylindole (DAPI), as described previously [29]. The experiments were repeated three times independently. A total of 150 hyphae were examined for each strain.

For conidiation assays, two methods were used. One method used was to count spores produced in CMC liquid media, using fresh mycelia (50 mg) of each strain taken from the edge of a 3-day-old colony to inoculate 100 ml of CMC liquid media. The flasks were incubated at 25°C for 5 days in a shaker (180 rpm). The other method used was to count spores produced on MBA, using 2 μl of a 1*10^6^ ml^−1^ spore suspension to inoculate a MBA plate. After one week of incubation, two ml of water was added to the plate and spread over the mycelium to collect spores. The amount of conidia was counted with a hemacytometer. The experiments were repeated three times independently. There are three replicates for each experiment.

Additionally, conidia of each strain were re-suspended in 2% (w/v) sucrose solutions and incubated at 25°C for 4 hrs, then conidial germination was examined under a Nikon ECLIPSE Ni-U microscope. Conidial morphology was observed under the same microscope. Furthermore, septum and nucleus were examined after staining conidia from each strain with calcofluor white (CFW) and DAPI, respectively, as described previously [29]. The experiments were repeated three times independently. A total of 150 conidia were examined for each strain.

### Sensitivity determination to different stress agents

To determine the sensitivity to different temperatures, 5-mm mycelial plugs of each strain taken from 3-day-old colony edge were inoculated on PDA and then incubated at 4, 10, 15, 20, and 25°C. Three replicate plates for each temperature were used for each strain. After incubation for 3.5 days, colony diameter in each plate was measured in two perpendicular directions with the original mycelial plug diameter (5 mm) subtracted from each measurement. For each plate, the average of the colony diameters was used for calculating the percentage of growth inhibition. The mycelium growth of each strain at 25°C was arbitrarily set as control. The experiment was repeated three times. There are two replicates for each experiment.

To determine the sensitivity to various stresses, 5-mm mycelial plugs of each strain taken from 3-day-old colony edge were inoculated on MM amended with antifungal compound carbendazim, tebuconazole, iprodione or fludioxonil, on fructose gelatin agar (FGA; 10 g fructose, 2 g gelatin, 1 g KH_2_PO_4_, 0.5 g MgSO_4_°7H_2_O, 2 g NaNO_3,_ 20 g agar and 1 l water, pH 7.0) amended with antifungal compound pyrimethanil, or on MM amended with cell wall stress agent congo red or SDS, osmotic agent NaCl or sorbitol, oxidative stress generator H_2_O_2_ or paraquat, or metal ion CaCl_2_ or MnCl_2_. The fungicides described above were kindly provided by the Institute of Zhejiang Chemical Industry or the Institute for the Control of Agrochemicals, Ministry of Agriculture (ICAMA), Beijing, China. The concentration of each compound is presented in Additional file 2. After incubation for 3.5 days, colony diameter in each plate was measured in two perpendicular directions with the original mycelial plug diameter (5 mm) subtracted from each measurement. For each plate, the average of the colony diameters was used for calculating the percentage of growth inhibition. The experiment was repeated three times. There are two replicates for each experiment.

### Pathogenicity assays on flowering wheat heads and tomatoes

Pathogenicity test was performed with single floret injection method as previously described [30]. After incubation in CMC medium for 5 days, conidia of each strain were collected by filtration through three layers of gauze and subsequently resuspended in sterile distilled water to a concentration of 1 × 10^5^ conidia ml^−1^. A 10-μl aliquot of conidial suspension was injected into a floret in the central section spikelet of single flowering wheat heads of susceptible cultivar Jimai 22. There were ten replicates for each strain. After inoculation, the plants were kept at 22 ± 2°C under 95–100% humidity with 12 hrs of daylight. Fifteen days after inoculation, the infected spikelets in each inoculated wheat head were recorded. The experiment was repeated four times. To examine the ability to colonize tomato, a 10-μl aliquot of conidial suspension was injected into the wounded tomato after surface sterilization. There are five replicates for each strain. Inoculated tomatoes were incubated under the same conditions described above, and were photographed 3 days after inoculation. The experiment was repeated three times.

### Determination of DON production

A 50-g aliquot of healthy wheat kernels was sterilized and inoculated with 50 mg fresh mycelia of each strain. After incubation at 25°C for 20 days, DON and fungal ergosterol were extracted using previously described protocols [31]. The DON extracts were purified with PuriToxSR DON column TC-T200 (Trilogy analytical laboratory), and amounts of DON and ergosterol in each sample were determined by using a HPLC system Waters 1525. The experiment was repeated three times, each with three replicates.

### Analysis of gene expression

Total RNA of each strain was extracted using the TaKaRa RNAiso Reagent, and 10 μg of each RNA sample was used for reverse transcription with the oligo(dT)_18_ primer using a RevertAid H Minus First Strand cDNA Synthesis kit. Expression of each gene was determined by quantitative reverse-transcriptase PCR (RT-PCR) with the corresponding primer pair (Additional file 1). The RT-PCR amplifications were performed in a DNA Engine Opticons 4 System (MJ Research) using the SYBR Green I fluorescent dye detection. Amplifications were conducted in 20-μl volumes containing 10 μl SYBR® Premix Ex Taq (TaKaRa Biotechnology Co., Ltd, Dalian, China), 2 μl template DNA and 1 μl each 4 μM primer (each primer pair per amplification). There were three replicates for each sample. The real-time PCR amplifications were performed with the following parameters: an initial preheat at 95°C for 2 min, followed by 35 cycles at 95°C for 15 s, 58°C for 20 s, 72°C for 20 s, and 80°C for 3 s in order to quantify the fluorescence at a temperature above the denaturation of primer-dimers. Once amplifications were completed, melting curves were obtained to identify PCR products. For each sample, PCR amplifications with primer pair Actin-F + Actin-R (Additional file 1) for the quantification of expression of *ACTIN* gene were performed as a reference. The relative expression levels of each gene in each strain or under the treatment were calculated using the 2^-ΔΔCt^ method [32]. The experiment was repeated three times.

### Standard molecular methods

Fungal genomic DNA was extracted using a previous published protocol [33]. Plasmid DNA was isolated using a Plasmid Miniprep Purification Kit (BioDev Co., Beijing, China). Southern analysis of *FaTBCA* gene in *F. asiaticum* was performed using probe as indicated in Additional file 3. The probe was labeled with digoxigenin (DIG) using a High Prime DNA Labeling and Detection Starter kit II according to the manufacturer’s instructions (Roche Diagnostics; Mannheim, Germany).

### Statistical analysis

All data for gene expression, DON production, conidiation and conidial germination were subjected in an analysis of variance (ANOVA) and the means were separated using Fisher’s protected least significant difference (*P* = 0.05). In the sensitivity to environmental stresses assays, the mycelial growth inhibition of ΔFaTBCA-3 under each stress was compared with that of the wild type using a *t* test.

## Results

### *In silico* analysis of FaTBCA in *F. asiaticum*

One tubulin binding cofactor A (TBCA) (FGSG_00510.3) was retrieved by BLASTP searching the *F. graminearum* genome database (http://www.broadinstitute.org) with the *S. cerevisiae* TBCA orthologue Rbl2 as a query. Based on the DNA sequences of FGSG_00510.3, the corresponding orthologue *FaTBCA* was amplified and sequenced from *F. asiaticum*. Sequencing analysis showed that the nucleotide sequence of *FaTBCA* is 425 bp in length including one predicted intron, and is predicted to encode a 119 amino-acid protein. Phylogenic analyses showed that FaTBCA is homologous to their counterparts from other filamentous fungi and yeast (Additional file 4). In addition, protein domain analysis by Pfam (http://pfam.xfam.org/) revealed that FaTBCA contains a conserved TBCA domain (Additional file 4). However, the deduced amino acid sequence of TBCA from the wild-type GJ33 is not highly homologous to those from other fungi (Additional file 4). For example, the deduced amino acid sequence of TBCA in GJ33 shared 46.0, 37.7 and 30.3% identity to those from *Magnaporthe oryzae* (MagoCoA, XP_003709983.1), *Aspergillus nidulans* (AnCoA, CBF70033.1) and *S. cerevisiae* (SaCoA, EGA72990.1), respectively.

### FaTBCA partially complement the yeast ΔRbl2 mutant

**Figure 1:**
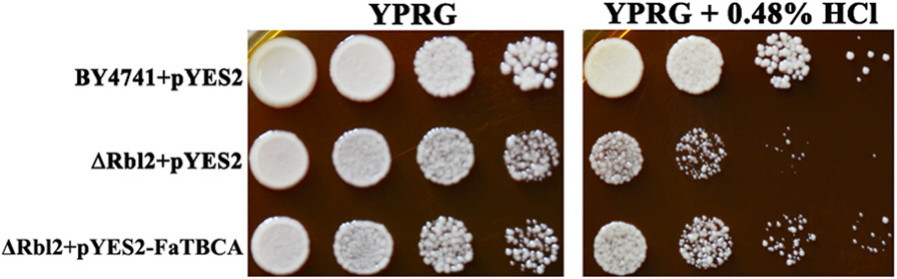
***Fusarium asiaticum FaTBCA*** **restored HCl sensitivity of the yeast ΔRbl2 mutant.** Cells of the mutant containing pYES2 or pYES2-FaTBCA were spotted onto YPRG supplemented with 0.48% (v/v) HCl. The wild-type BY4741 transformed with empty pYES2 was used as a control.

To characterize the functions of *FaTBCA*, we first tested whether this gene could complement an HCl-sensitive *S. cerevisiae TBCA* mutant (ΔRbl2) whose growth is dramatically inhibited on YPRG amended with 0.48% (v/v) HCl. An expression vector pYES2 containing the full-length *FaTBCA* cDNA was transferred into ΔRbl2. As a negative control, the mutant was also transformed with an empty pYES2 vector. As shown in Figure 1, the reduced growth of yeast ΔRbl2 on YPRG +0.48% HCl was partially restored by *F. asiaticum FaTBCA.* These results indicate that FaTBCA could function as a TBCA in *S. cerevisiae*.

### Deletion and complementation of *FaTBCA* in *F. asiaticum*

To investigate the function of *FaTBCA*, we generated gene deletion mutants using a homologous recombination strategy (Additional file 3). Seven deletion mutants were identified from 13 hygromycin-resistant transformants by PCR analysis with the primer pair A9 + A10 (Additional file 1). The primer pair amplified 1710 and 460 bp fragments from *FaTBCA* deletion mutants and the wild-type progenitor GJ33, respectively. All seven deletion mutants showed identical growth defects on PDA plates. When probed with a 973 bp upstream DNA fragment of *FaTBCA*, the deletion mutant ΔFaTBCA-3 had an anticipated 5315 bp band, but lacked a 2205 bp band which was present in the wild-type GJ33 (Additional file 3). This Southern hybridization pattern confirmed that the transformant ΔFaTBCA-3 is a null mutant resulting from expected homologous recombination events at the *FaTBCA* locus. The complemented strain ΔFaTBCA-3C was a single copy of *FaTBCA* inserted into the genome of ΔFaTBCA-3 (Additional file 3).

### Involvement of FaTBCA in vegetative growth and conidiation in *F. asiaticum*

**Figure 2:**
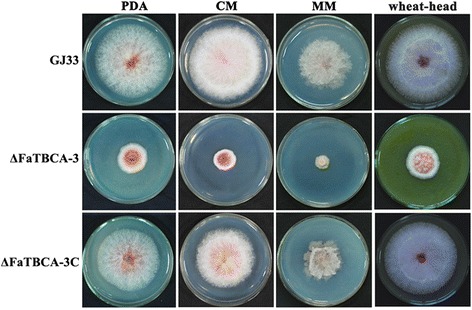
**Impact of** ***FaTBCA*** **deletion on** ***F. asiaticum*** **hyphal growth.** The wild-type GJ33, ΔFaTBCA-3 and ΔFaTBCA-3C were grown on solid media, PDA, CM, MM and wheat-head at 25°C for 3 days.

**Figure 3:**
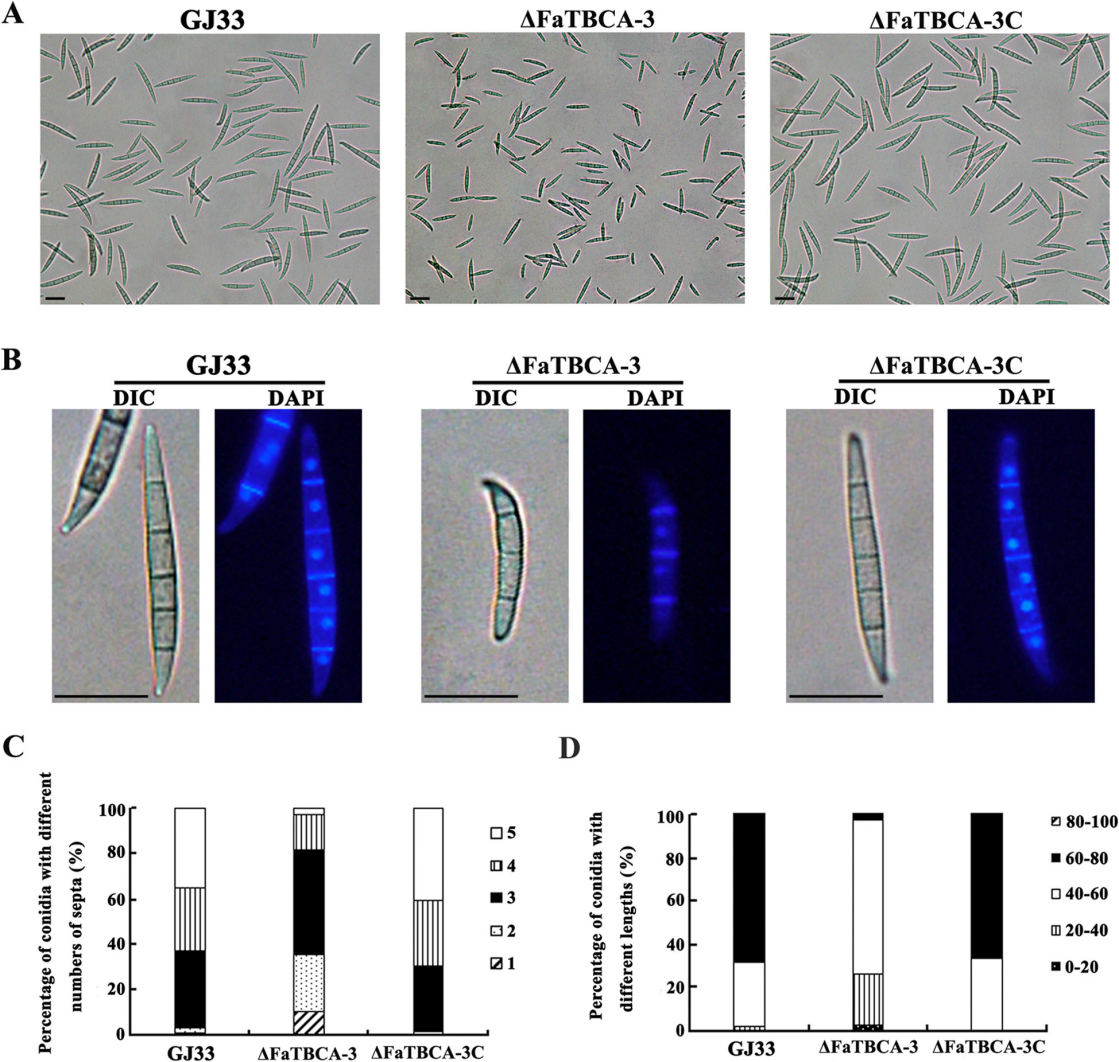
**Involvement of** ***FaTBCA*** **in regulation of conidial morphology in** ***F. asiaticum*** **. A**. Compared with the wild-type GJ33, ΔFaTBCA-3 produced shorter conidia with less septation. The differential interference contrast [DIC] images of conidia were captured with an electronic microscope. Bar = 40 μm. **B**. Conidial morphology of GJ33, ΔFaTBCA-3 and ΔFaTBCA-3C. DIC images of conidia stained with 4',6-diamidino-2-phenylindole (DAPI) were captured with an electronic microscope. Bar = 40 μm. **C**. Comparisons of the percentage of conidia with different septum numbers among GJ33, ΔFaTBCA-3 and ΔFaTBCA-3C. A total of 150 conidia were examined for each strain. **D**. Comparisons of conidial length among GJ33, ΔFaTBCA-3 and ΔFaTBCA-3C. A total of 150 conidia were examined for each strain.

The *FaTBCA* deletion mutant ΔFaTBCA-3 grew significantly slower than the wild-type GJ33 on PDA, CM, MM and wheat-head media (Figure 2). Compared to the wild-type GJ33, ΔFaTBCA-3 produced a similar number of conidia (Additional file 5), however, conidia of ΔFaTBCA-3 were shorter and with less septa (Figure 3A and B). Microscopic examination showed that 81.7% of ΔFaTBCA-3 conidia had one to three septa with conidial lengths ranging from 23.6 to 57.7 μm (Figure 3A, C and D). In contrast, 62.6% of the wild-type conidia had four or five septa, with lengths ranging from 62.7 to 80.4 μm (Figure 3A, C and D). In addition, conidial germination assays found that conidia of ΔFaTBCA-3 did not show delayed germination (Additional file 5).

### The *FaTBCA* deletion mutant showed increased sensitivity to low temperatures

**Figure 4:**
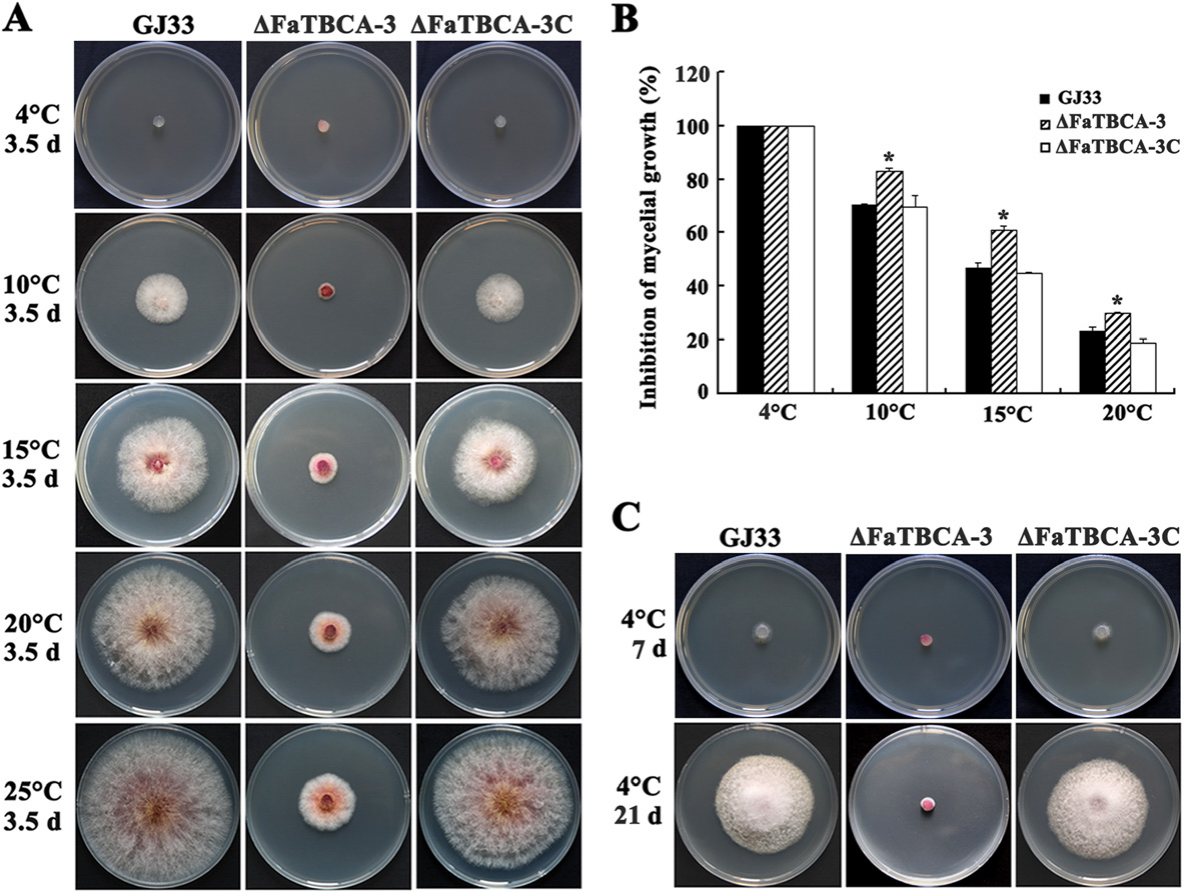
**The deletion of** ***FaTBCA*** **increased sensitivity to low temperatures. A**. Mycelial plugs of the wild-type GJ33, ΔFaTBCA-3 and ΔFaTBCA-3C were inoculated on PDA and incubated at 4, 10, 15, 20 and 25°C for 3.5 days. **B**. Inhibition percentages of mycelial growth of GJ33, ΔFaTBCA-3 and ΔFaTBCA-3C at 4, 10, 15 and 20°C. After incubation for 3.5 days, colony diameter in each plate was measured in two perpendicular directions with the original mycelial plug diameter (5 mm) subtracted from each measurement. For each plate, the average of the colony diameters was used for calculating the percentage of growth inhibition. The mycelium growth of each strain at 25°C was arbitrarily set as control. Line bars in each column denote standard errors of three experiments. * = significant difference for each temperature at a 95% coincidence interval by performing a *t* test. **C**. The mutant ΔFaTBCA-3 was unable to grow at 4°C after incubation for 7 or 21 days.

Previous studies have reported that cold temperatures have disruptive effects on the stability of microtubules [34,35], we therefore determined the sensitivity of the *FaTBCA* deletion mutant to low temperatures. As shown in Figure 4A and B, the inhibition percentages of mycelial growth of the mutant ΔFaTBCA-3 at 10, 15 and 20°C were significantly higher than those of the wild type. Furthermore, after 7 days of incubation at 4°C, the wild type exhibited an obvious growth, however, ΔFaTBCA-3 did not show any sign of growth (Figure 4C). After 21 days of incubation at 4°C, the colony diameter of the wild type was greater than 5.8 cm, whereas, the ΔFaTBCA-3 mutant was still unable to grow (Figure 4C), indicating that the deletion of *FaTBCA* caused the stagnation of growth at 4°C. The ΔFaTBCA-3 mutant restored growth after shifting from 4 to 25°C (data not shown).

**Figure 5:**
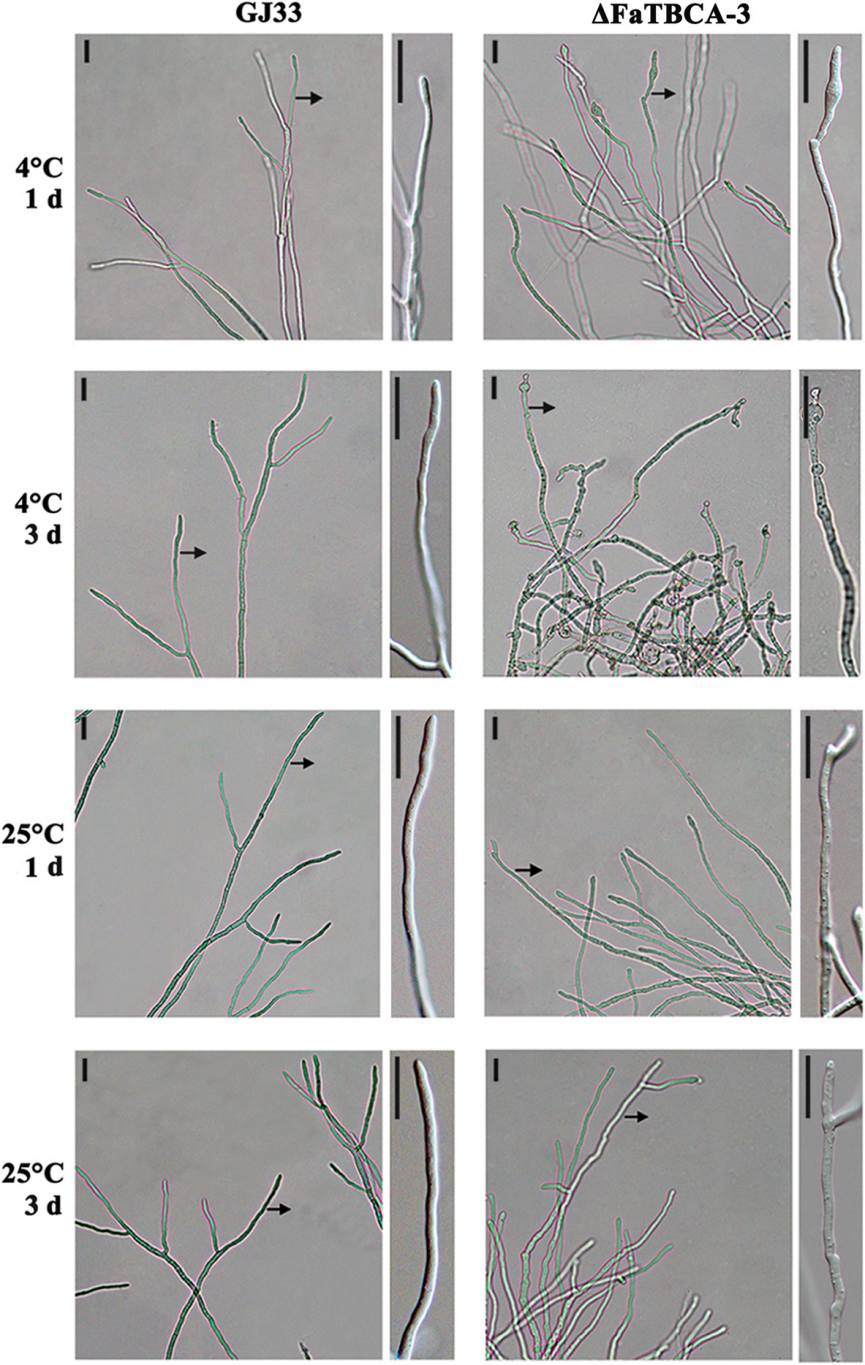
**Hyphae of ΔFaTBCA-3 exhibit blebbing phenotypes under cold stress.** Differential interference contrast [DIC] images of hyphae were captured with an electronic microscope. Bar = 100 μm. The enlarged hyphae are indicated by black arrows. The wild-type GJ33 and ΔFaTBCA-3 were incubated in PDB at 25°C for 1 day with a shaker then transferred to static incubation at 4 and 25°C for 1 or 3 days.

**Figure 6:**
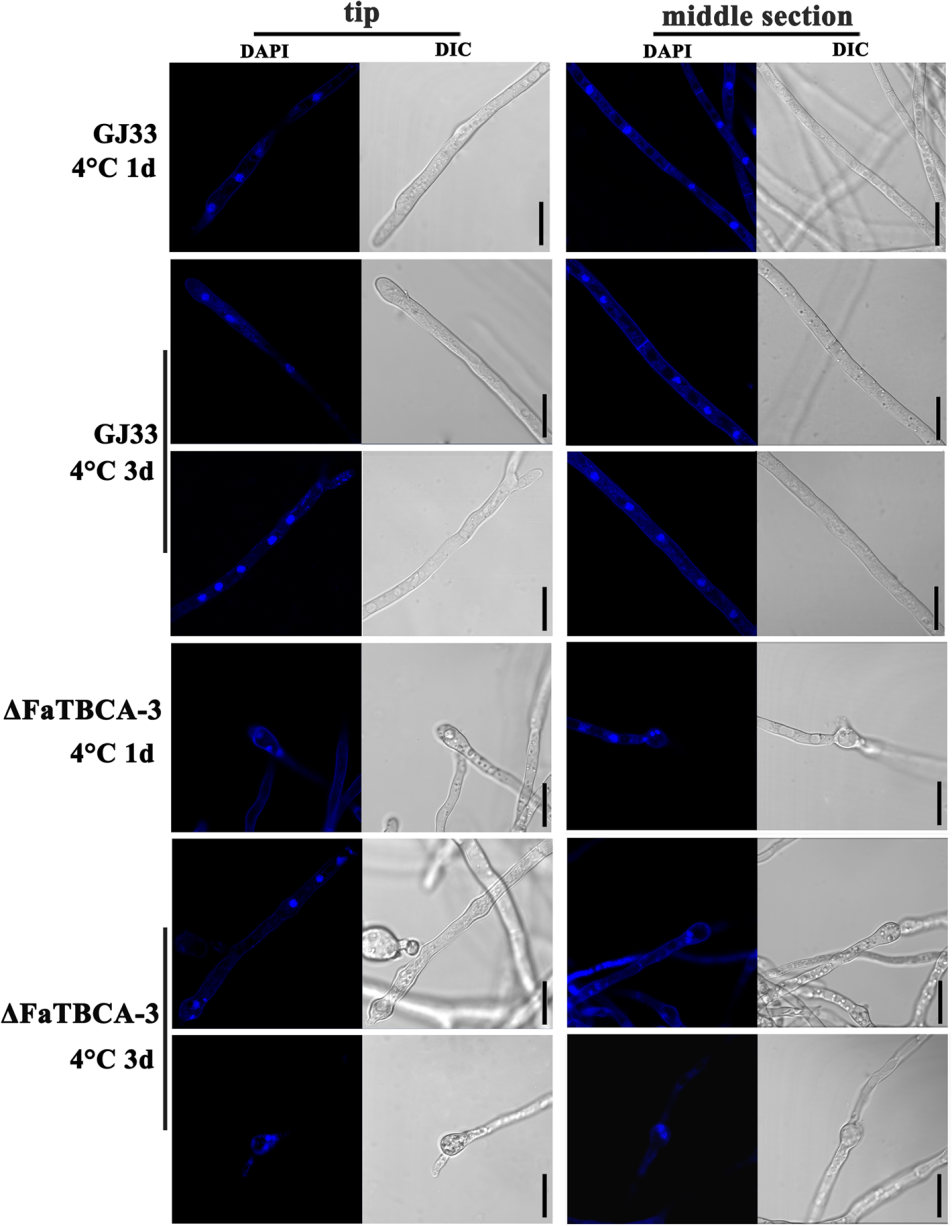
**The number of nuclei in ΔFaTBCA-3 increases under cold stress.** Differential interference contrast [DIC] images of nuclei in hyphal tip and middle section of the wild-type GJ33 and ΔFaTBCA-3 were taken after staining with 4',6-diamidino-2-phenylindole (DAPI). Bar = 10 μm. Each strain was incubated in PDB at 25°C for 1 day with a shaker then transferred to static incubation at 4°C for 1 or 3 days.

In order to explore why the mutant ΔFaTBCA-3 cannot grow at the cold temperature 4°C, we further observed the hyphal morphology and nuclear distribution of ΔFaTBCA-3 after 1-day or 3-day incubation at 4°C. After incubation in a shaker at 25°C for 1 day, the wild type and ΔFaTBCA-3 were immediately transferred to 4°C for 1 or 3 days. Microscopic observation showed that the hyphal tips of ΔFaTBCA-3 began to present blebbing phenotypes after 1-day incubation at 4°C (Figure 5). Moreover, the enlarged nodes occurred not only in hyphal tips but also in hyphal middle sections after 3-day incubation (Figure 5). By observing 150 hyphal tips, we found that 9 and 83% hyphal tips showed bulged nodes after 1 and 3 days of incubation at 4°C, respectively. Furthermore, 4',6-diamidino-2-phenylindole (DAPI) staining assays showed that approximately 72% enlarged nodes contained several nuclei by counting 100 enlarged nodes (Figure 6). However, the hyphal morphology and nuclear distribution of the wild type after incubation at 4°C exhibited no obvious difference compared to those after incubation at 10 or 25°C (Figure 5, 6and Additional file 6). Taken together, these results indicate that *FaTBCA* plays a critical role in the cell division under cold stress (4°C) in *F. asiaticum*.

### The deletion of *FaTBCA* did not change the sensitivity to carbendazim

Since the fungicide carbendazim can cause the depolymerization of fungal microtubules, we tested the sensitivity of the mutant to this compound. Surprisingly, ΔFaTBCA-3 did not differ from the wild type in carbendazim sensitivity, as well as in other fungicides including tebuconazole, iprodione, fludioxonil and pyrimethanil (Additional file 2). In addition, the deletion of *FaTBCA* had no effect on the sensitivity of *F. asiaticum* to cell wall damaging agents, and to osmotic, oxidative and metal cation stresses (Additional file 2).

Given that the *FaTBCA* deletion mutant ΔFaTBCA-3 did not show increased sensitivity to carbendazim, we were interested in the expression of *FaTBCA* treated with carbendazim and the expression of two α-tubulin genes (encoded by *FaTUA1* and *FaTUA2*) in ΔFaTBCA-3 mutant. As shown in Additional file 7, the expression of *FaTBCA* increased 3.1 times under the treatment of 3.5 μg ml^−1^ carbendazim. Quantitative real-time PCR analysis showed that the expression of *FaTUA1* and *FaTUA2* in ΔFaTBCA-3 showed no significant difference compared with the wild-type GJ33 (Additional file 7), indicating that there might be other unknown genes involved in regulating the balance of the α/β-tubulin monomers.

### FaTBCA is required for full virulence and DON biosynthesis in *F. asiaticum*

**Figure 7:**
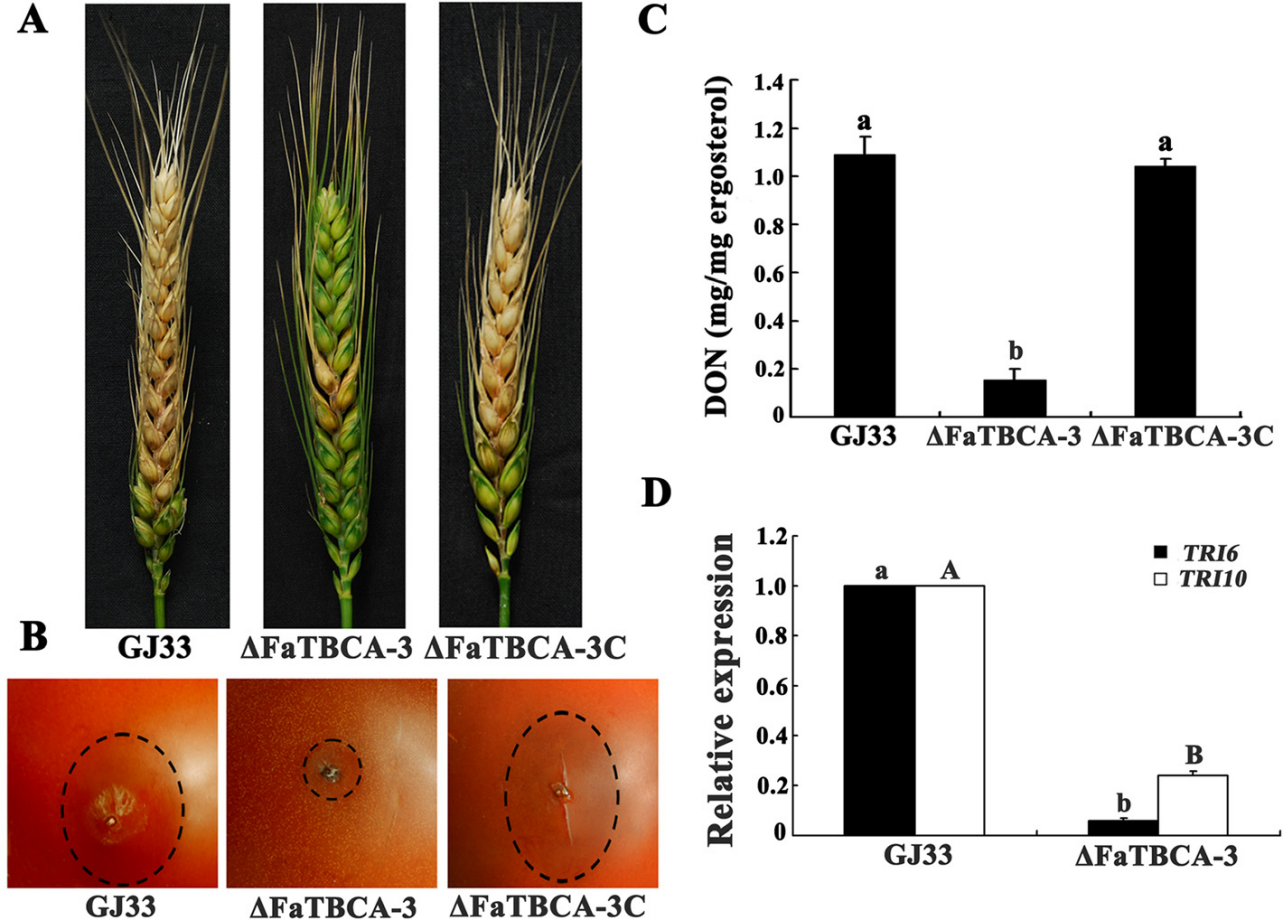
**FaTBCA is required for full virulence and DON biosynthesis in** ***F. asiaticum*** **. A**. Flowering wheat heads were point inoculated with a conidial suspension at 10^5^ conidia ml^−1^ of the wild-type GJ33, ΔFaTBCA-3 and ΔFaTBCA-3C, and infected wheat heads were photographed 15 days after inoculation. **B**. Tomatoes were inoculated with a conidial suspension at 10^5^ conidia ml^−1^ of each strain and infected fruits were photographed 3 days after inoculation. **C**. The amount of DON (per mg fungal ergosterol) produced by each strain in infected wheat kernels was determined after 20 days of inoculation. Line bars in each column denote standard errors of three experiments. Values on the black bars followed by the same letter are not significantly different according to a Fisher’s least significant difference (LSD) test at *P* = 0.05. **D**. Relative expression of DON biosynthetic *TRI* genes in each strain and bars denote standard errors from three experiments. Values on the black bars followed by the same letter for each gene are not significantly different according to a Fisher’s least significant difference (LSD) test at *P* = 0.05.

Pathogenicity assays indicated that disruption of *FaTBCA* caused a significant reduction in the virulence of *F. asiaticum*. As shown in Figure 7A, the wild-type strain GJ33 and ΔFaTBCA-3C caused the typical scab symptoms in the inoculated and nearby spikelets of flowering wheat heads 15 days after inoculation. Under the same conditions, however, scab symptoms caused by ΔFaTBCA-3 were only observed in the inoculated spikelet and in the adjacent two or three spikelets. Furthermore, three days after inoculation of non-host tomatoes, the water-soaked rot lesion caused by ΔFaTBCA-3 was significantly smaller than that caused by GJ33 or ΔFaTBCA-3C (Figure 7B).

Previous studies have shown that DON is an important virulence factor and a prerequisite for colonization of wheat [36,37]. Therefore, we were interested in the effect of *FaTBCA* deletion on DON biosynthesis. After growth on sterilized wheat kernels for 20 days, the amount of DON produced by the wild type was 6.2 folds higher than that produced by ΔFaTBCA (Figure 7C). To further confirm the results, we determined the expression levels of *TRI6* and *TRI10* by quantitative real-time PCR using RNA samples isolated from mycelia grown in minimal synthetic liquid medium (MS) [38] for 4 days at 25°C. The expression levels of *TRI6* and *TRI10* in ΔFaTBCA reduced by 94% and 76%, respectively, as compared to those in the wild type (Figure 7D). These results indicate that FaTBCA plays a critical role in DON biosynthesis in *F. asiaticum*.

## Discussion

In this study, we characterized a putative tubulin cofactor A FaTBCA, a homologue of *S. cerevisiae* Rbl2 in *F. asiaticum*. Although FaTBCA shares low homology with similar proteins found in other organisms, *F. asiaticum* FaTBCA can restore the sensitivity to HCl of the *RBL2* deletion mutant of budding yeast. Similarly, expression of mouse TBCA not only suppresses the benomyl supersensitivity resulting from the deletion of *RBL2* in *S. cerevisiae* [7], but also rescues all defects of a *KIS* (*TBCA* orthologue) mutant under the control of the 35S promoter in Arabidopsis [9]. The protein sequence of mouse TBCA is approximately 30% and 35% identical to Rbl2 and Kis, respectively. Therefore, the functions of TBCA homologues from yeast to higher eukaryotic organisms are evolutionally conserved.

Although TBCA in eukaryotic organisms are conserved, the detailed roles of TBCAs among different species are not fully consistent. In this present study, the deletion of *FaTBCA* leads to reduced vegetative growth and abnormal conidia with less septation in *F. asiaticum* (Figures 2 and 3), which is not in agreement with the situations in yeast. In *S. cerevisiae* and *S. pombe*, TBCA homologues are dispensable for cell growth and asexual development, but played important roles in meiosis and growth polarity, respectively. Previous studies on *F. asiaticum* reported that the deletion of each β-tubulin results in reduced vegetative growth, abnormal conidial morphology or decreased conidiation [21,39,40]. Our *FaTBCA* deletion mutant ΔFaTBCA-3 shared similar phenotypes with β-tubulin disruption mutants of *F. asiaticum*, indicating that TBCA in *F. asiaticum* is involved in the maintenance of microtubule function as reported in other eukaryotic organisms.

Microtubules are dynamic by nature, with an equilibrium existing between soluble subunits and the polymerized filament that could influence normal cellular functions. Previous studies have found that low temperature generally shifts this equilibrium toward depolymerization and leads to intrinsic cold sensitivity of microtubule [34,35]. In this study, we found that the deletion of *FaTBCA* increased the sensitivity to low temperatures in *F. asiaticum* (Figure 4A and B), indicating that deletion of *FaTBCA* might accelerate the depolymerization of microtubule at low temperatures. However, a precise monitoring of microtubule dynamics under cold stress would have to be addressed to confirm this hypothesis.

Under the cold temperature 4°C, the *FaTBCA* mutant exhibited growth stagnation accompanied with the phenotype of several nuclei in the enlarged hyphal nodes (Figures 5 and 6), indicating that *FaTBCA* is involved in cell division. This might be a major contributor to the stagnation of growth at 4°C. In Arabidopsis, mesophyll cells in leaf sections of *KIS* mutant frequently were highly enlarged and the enlarged cells had one large nucleus or several nuclei [9]. In human HeLa cells transfected with TBCA siRNA also exhibits blebbing phenotypes, which are considered to be due to the changes of microtubule structures [10]. Taken together, our results showed that the TBCA in *F. asiaticum* might share some functions with Kis in Arabidopsis [9].

Carbendazim and other benzimidazole fungicides, which target β-tubulin and interrupt the polymerization of microtubules, have been extensively used to control various plant diseases caused by fungi [40]. The treatment of anti-microtubule drug carbendazim caused the up-regulation of *FaTBCA* in *F. asiaticum* (Additional file 7), which supported that TBCA serves as a reservoir of excess β-tubulin [1,8]. To our surprise, unlike the case in *S. cerevisiae* [7], the absence of *FaTBCA* did not render cells more sensitive to carbendazim (Additional file 2). In budding yeast, over-expression of either Rbl2 or α-tubulin suppresses β-tubulin lethality and causes resistance to antimicrotubule drug benomyl [7]. However, our quantitative real-time PCR assays found that the deletion of *FaTBCA* did not result in significant change in the expression levels of two α-tubulin genes (Additional file 7), indicating that there might be other unknown genes involved in the control of the α/β-tubulin monomer balance.

In addition to the involvement of FaTBCA in regulating mycelial growth, conidiation and low temperature sensitivity, this gene is also required for virulence of *F. asiaticum*. The reduced virulence in ΔFaTBCA may result from three defects of the mutant. First, ΔFaTBCA showed drastic mycelium growth defect, which might be a major contributor. As observed on PDA, the mutant also grew significantly slower than the wild-type strain on the wheat-head medium (Figure 2). Second, the ability of ΔFaTBCA to produce trichothecene mycotoxins in infected wheat kernels was greatly decreased. DON, the end product of the trichothecene biosynthetic pathway, plays an important role in the spread of FHB within a spike [36,37], thus this may also contribute to the reduced virulence of the mutant. Third, TBCA has been found to play a crucial role in correct polymerization of microtubules [7,9]. Moreover, previous studies have reported that extracellular secretion of virulence factors is dependent on microtubule-mediated vesicle transport in pathogenic fungi [41-43]. Thus, the reduced virulence in ΔFaTBCA-3 might be associated with the disturbance of microtubule-dependent vesicle transport.

## Conclusion

Our *FaTBCA* studies in *F. asiaticum* found that FaTBCA plays critical roles in vegetative growth, conidial morphology, sensitivity to low temperatures and virulence. To our knowledge, this is the first report about the functions of TBCA in filamentous fungi. Our results indicate that the functions of TBCA in *F. asiaticum* are partially different from what have reported in yeast.

## Additional files

Additional file 1:
**Oligonucleotide primers used in this study.**
Additional file 2:
**Sensitivity of GJ33, ΔFaTBCA-3 and ΔFaTBCA-3C to environmental stresses.** A. Comparisons were made on MM plates amended with 10 μg ml^−1^ iprodione, 0.25 μg ml^−1^ tebuconazole, 0.5 μg ml^−1^ carbendazim, 0.05 μg ml^−1^ rapamycin, 0.25 M CaCl_2_, 10 μg ml^−1^ paraquat, 0.05% H_2_O_2_, 0.7 M NaCl, 0.7 M sorbitol, 0.01% SDS, and 0.2 g l^−1^ congo red. B. Inhibition percentages of mycelial growth of GJ33, ΔFaTBCA-3 and ΔFaTBCA-3C under each stress. After incubation for 3.5 days, colony diameter in each plate was measured in two perpendicular directions with the original mycelial plug diameter (5 mm) subtracted from each measurement. For each plate, the average of the colony diameters was used for calculating the percentage of growth inhibition. Line bars in each column denote standard errors of three experiments. A *t* test was performed to determine significant differences, * = significant difference for each stress at a 95% coincidence interval. Additional file 3:
**Generation and identification of** ***FaTBCA*** **gene deletion mutant.** A. Target gene *FaTBCA* deletion strategy. The hygromycin resistance cassette (*HPH*) is denoted by the large gray arrow. Primer binding sites are indicated by arrows (see Additional file 1 for the primer sequences). B. DNA hybridization analysis of the wild-type strain GJ33, *FaTBCA* deletion mutant ΔFaTBCA-3, and the complemented transformant ΔFaTBCA-3C, using a 973 bp *FaTBCA* fragment as a probe. Genomic DNA of each strain was digested with *Hin*dIII. Additional file 4:
**A. Phylogenetic analysis of tubulin cofactor A (TBCA) orthologues from** ***Fusarium asiaticum*** **(FaTBCA, KM116518; indicated in the black boxes),** ***F. graminearum*** **(FgTBCA, FGSG_00510.3),** ***F. pseudograminearum*** **(FpTBCA, EKJ75396.1),** ***F. verticillioides*** **(FvTBCA, EWG37294.1),** ***F. fujikuroi*** **(FfTBCA, CCT62423.1),** ***F. oxysporum*** **(FoTBCA, EWY93729.1),** ***Claviceps purpurea*** **(CpTBCA, CCE31346.1),** ***Colletotrichum graminicola*** **(CgTBCA, EFQ26205.1),** ***Co. orbiculare*** **(CoTBCA, ENH78242.1),** ***Verticillium dahliae*** **(VdTBCA, EGY18367.1),** ***Togninia minima*** **(TmTBCA, XP_007910919.1),** ***Neurospora crassa*** **(NcTBCA, XP_964483.1),** ***Magnaporthe oryzae*** **(MoTBCA, XP_003709983.1),** ***Gaeumannomyces graminis*** **(GgTBCA, EJT76136.1),** ***Marssonina brunnea*** **(Mb510, XP_007289220.1),** ***Blumeria graminis*** **(BgTBCA, CCU76202.1),** ***Neofusicoccum parvum*** **(NpTBCA, XP_007583539.1),** ***Aspergillus nidulans*** **(AnTBCA, CBF70033.1),** ***Candida albicans*** **(CaTBCA, EEQ43562.1),** ***Saccharomyces cerevisiae*** **(ScRbl2, EGA72990.1), and** ***Schizosaccharomyces pombe*** **(SpAlp3, CAB40194.1).** The phylogenetic tree was generated by the neighbor-joining method with 1000 bootstrap replicates using the Mega 4.1 software. B. Each TBCA orthologue contains one functional domain, tubulin binding cofactor A, which were identified by Pfam (http://pfam.xfam.org/). C. Alignment of the predicted amino acid sequences of TBCA domains from *F. asiaticum*, *M. oryzae*, *N. crassa*, *A. nidulans*, *S. cerevisiae*, *S. pombe*, and *C. albicans*. The Boxshade program was used to highlight identical (black shading) or similar (grey shading) amino acids. Additional file 5:
**Effect of** ***FaTBCA*** **deletion on conidiation and conidium germination of** ***F. asiaticum.*** A. Conidia were counted after incubation of the wild-type GJ33, the mutant ΔFaTBCA-3 and the complemented strain ΔFaTBCA-3C in carboxymethyl cellulose liquid medium (CMC) for 5 days in a shaker at 25°C. B. Conidia were counted after incubation of each strain on mung bean agar (MBA) for one week of incubation at 25°C. C. Conidia of each strain were incubated in 2% (w/v) sucrose solutions and incubated at 25°C for 4 hrs. After incubation for 4 hrs, conidium germination of 150 conidia was examined. Line bars in each column denote standard errors of three repeated experiments. Bars with the same letter indicate no significant different according to a Fisher’s least significant difference (LSD) test at *P* = 0.05.T. Additional file 6:
**The number of nuclei in ΔFaTBCA-3 does not change after incubation at 10 or 25°C.** DIC images of nuclei in hyphal tip and middle section of the wild-type GJ33 and ΔFaTBCA-3 were taken after staining with 4',6-diamidino-2-phenylindole (DAPI). Bar = 10 μm. Each strain was incubated in PDB at 25°C for 1 day with a shaker then transferred to static incubation at 10 or 25°C for 3 days. Additional file 7:
**The relative expression levels of FaTBCA treated with 3.5 μg ml** ^−1^ **anti-microtubule drug carbendazim (A), FaTUA1 and FaTUA2 in the FaTBCA deletion mutant ΔFaTBCA-3 (B).** Line bars in each column denote standard errors of three experiments. Values on the black bars followed by the same letter for each gene are not significantly different according to a Fisher’s least significant difference (LSD) test at *P* = 0.05.
