# Supplementary material for: The tubulin cofactor A is involved in hyphal growth, conidiation and cold sensitivity in Fusarium asiaticum
Source: BMC Microbiol. 2015 Feb 18;15:35. doi: 10.1186/s12866-015-0374-z (PMC4342098; doi:10.1186/s12866-015-0374-z)
Supplement: Additional file 1: — Oligonucleotide primers used in this study. [file 12866_2015_374_MOESM1_ESM.doc]

**Table S1 Oligonucleotide primers used in this study and their relevant characteristics**

| **Name** | **Sequence(5’-3’)** | **Relevant characteristics** |
| --- | --- | --- |
| A1 | AGGCACCCAATTAACCGTTCT | PCR primers for amplification of the *FaTBCA* gene from *Fusarium asiaticum* strain GJ33 |
| A2 | TAATTCTTCCCTTCCCCCGT |
|  |  |  |
| A3 | ATggatccATGCCGCCGCCTTCACAACT | PCR primers for amplification of the full cDNA sequence of the *FaTBCA* gene for construction of FaTBCA-pYES2 vector |
| A4 | ATgaattcTTACGCATCGGTCTGAGTGG |
|  |  |  |
| A5 | ATctcgagCAGATGGTGTTGTAGGCGTTT | PCR primers for amplification of the upstream *FaTBCA* fragment |
| A6 | ATgtcgacGCGGCATGGTGAAATAGATT |
|  |  |  |
| A7 | ATaagcttGAGCAGACAAAGGGCGTTTT | PCR primers for amplification of the downstream *FaTBCA* fragment |
| A8 | ATggatccATGGAACCTTTTTTGTCCGC |
|  |  |  |
| A9 | ATTAACCGTTCTCGCTGCCTA | PCR primers for the identification of the *FaTBCA* disruption mutants |
| A10 | CACGTTGAGCTGGTCGCTTA |
|  |  |  |
| A11 | ATggtaccGAGATTGCCCTTTCAAGCAA | PCR primers for amplification of the entire *FaTBCA* gene including 1437 bp promoter region and 0 bp terminator region |
| A12 | ATtctagaTTACGCATCGGTCTGAGTGG |
|  |  |  |
| A13 | ACCACGGAGCAAACAAATCT | PCR primers to amplify the 973 bp *FaTBCA* fragment used as the probe for Southern blot analysis |
| A14 | CGGGGCTATTCCTGTCATCTA |
|  |  |  |
| A15 | TCAACCGCCTTCTCAAGGA | PCR primers for the quantitative analysis of *FaTBCA* expression |
| A16 | TTACAGCGAGCTGCTCCTCTA |
|  |  |  |
| Neo-F | ATctcgagGGAGGTCAACACATCAATGCT | PCR primers for amplification of geneticin resistance gene |
| Neo-R | ATggtaccTCAGAAGAACTCGTCAAGAAG |
|  |  |  |
| TuA1-F | TGTTTACCCTGCTCCTCAGA | PCR primers for quantitative analysis of *FaTUA1* expression |
| TuA1-R | TTGAGGGAACCGTCGAAT |
|  |  |  |
| TuA2-F | TCTCAACGAGTTCCAGACCAA | PCR primers for quantitative analysis of *FaTUA2* expression |
| TuA2-R | AGTCGCGAGGAACACAATCA |
|  |  |  |
| Actin-F | ATCCACGTCACCACTTTCAA | PCR primers for amplification of the reference gene *ACTIN* in quantitative real-time PCR assays |
| Actin-R | TGCTTGGAGATCCACATTTG |
