# Supplementary figures and images for: The tubulin cofactor A is involved in hyphal growth, conidiation and cold sensitivity in Fusarium asiaticum
Source: BMC Microbiol. 2015 Feb 18;15:35. doi: 10.1186/s12866-015-0374-z (PMC4342098; doi:10.1186/s12866-015-0374-z)

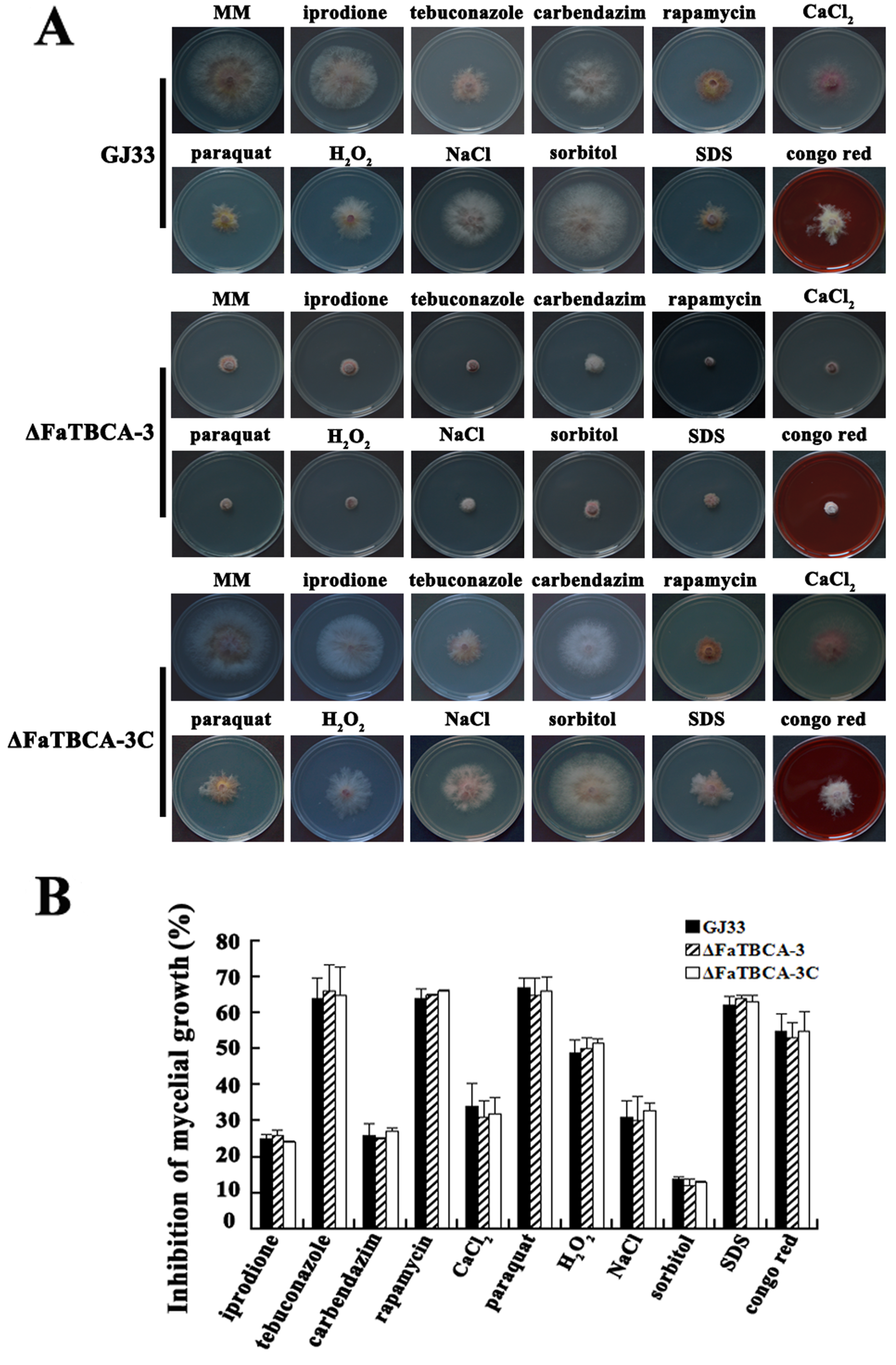

Supplement: Additional file 2: — Sensitivity of GJ33, ΔFaTBCA-3 and ΔFaTBCA-3C to environmental stresses. A. Comparisons were made on MM plates amended with 10 μg ml−1 iprodione, 0.25 μg ml−1 tebuconazole, 0.5 μg ml−1 carbendazim, 0.05 μg ml−1 rapamycin, 0.25 M CaCl2, 10 μg ml−1 paraquat, 0.05% H2O2, 0.7 M NaCl, 0.7 M sorbitol, 0.01% SDS, and 0.2 g l−1 congo red. B. Inhibition percentages of mycelial growth of GJ33, ΔFaTBCA-3 and ΔFaTBCA-3C under each stress. After incubation for 3.5 days, colony diameter in each plate was measured in two perpendicular directions with the original mycelial plug diameter (5 mm) subtracted from each measurement. For each plate, the average of the colony diameters was used for calculating the percentage of growth inhibition. Line bars in each column denote standard errors of three experiments. A t test was performed to determine significant differences, * = significant difference for each stress at a 95% coincidence interval. [file 12866_2015_374_MOESM2_ESM.tiff]

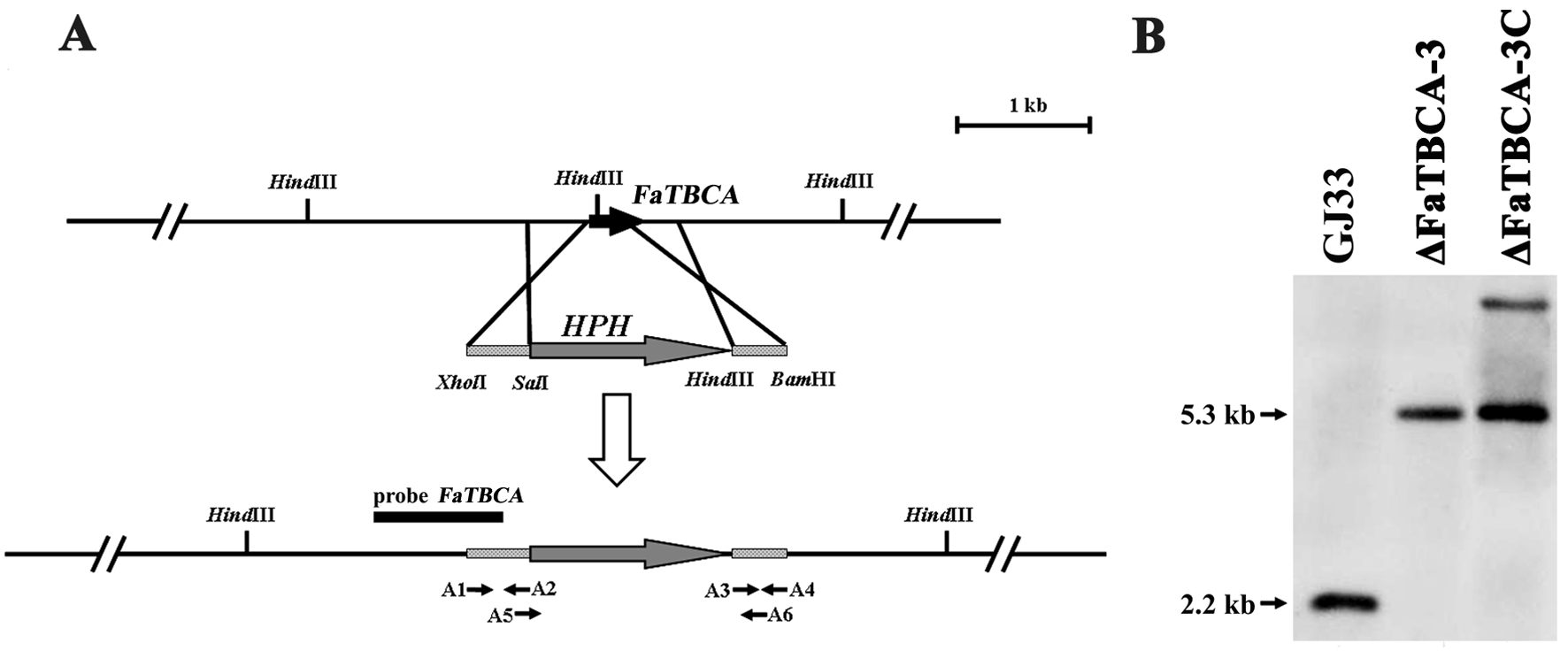

Supplement: Additional file 3: — Generation and identification of FaTBCA gene deletion mutant. A. Target gene FaTBCA deletion strategy. The hygromycin resistance cassette (HPH) is denoted by the large gray arrow. Primer binding sites are indicated by arrows (see Additional file 1 for the primer sequences). B. DNA hybridization analysis of the wild-type strain GJ33, FaTBCA deletion mutant ΔFaTBCA-3, and the complemented transformant ΔFaTBCA-3C, using a 973 bp FaTBCA fragment as a probe. Genomic DNA of each strain was digested with HindIII. [file 12866_2015_374_MOESM3_ESM.tiff]

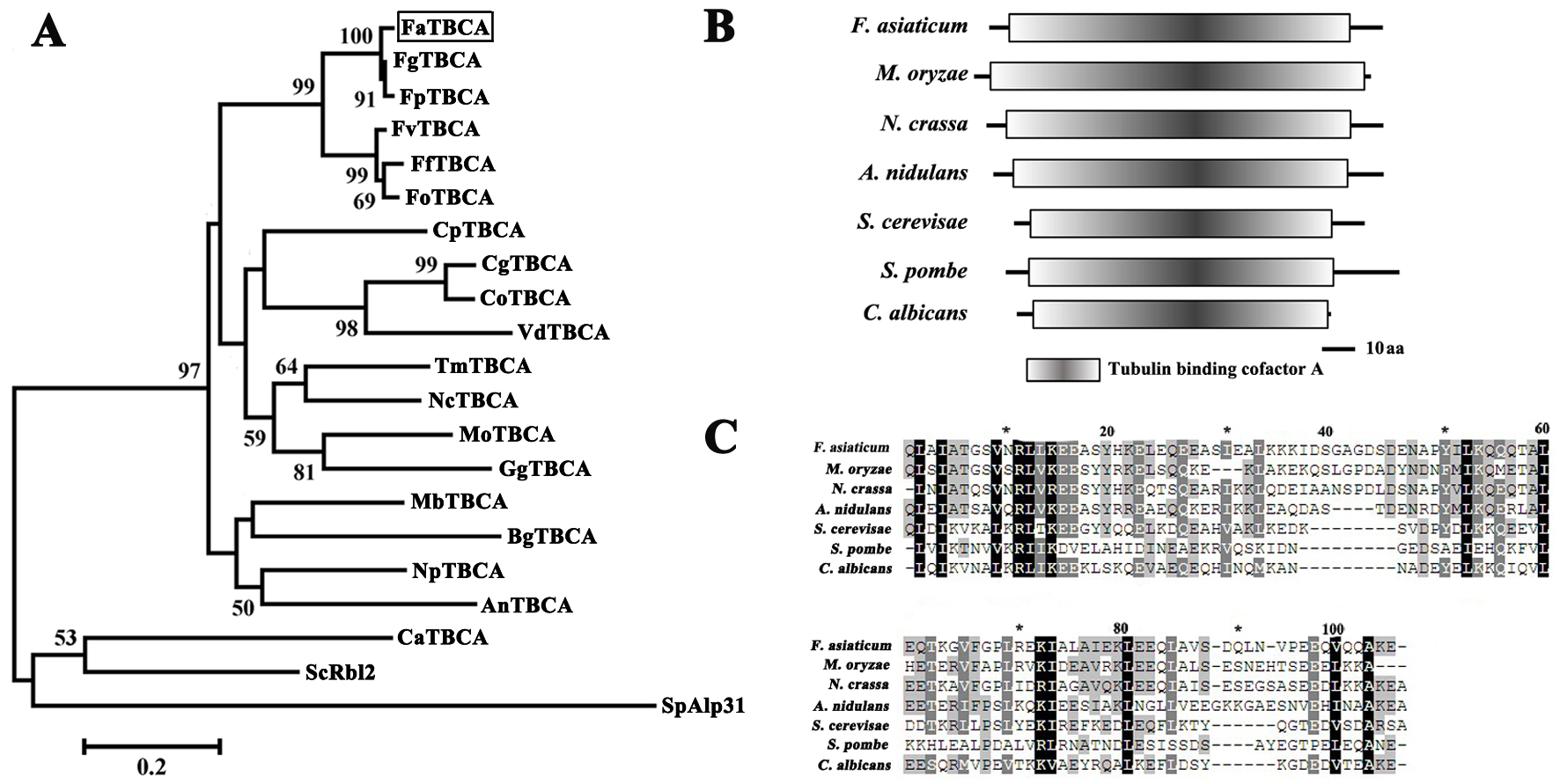

Supplement: Additional file 4: — A. Phylogenetic analysis of tubulin cofactor A (TBCA) orthologues from Fusarium asiaticum (FaTBCA, KM116518; indicated in the black boxes), F. graminearum (FgTBCA, FGSG_00510.3), F. pseudograminearum (FpTBCA, EKJ75396.1), F. verticillioides (FvTBCA, EWG37294.1), F. fujikuroi (FfTBCA, CCT62423.1), F. oxysporum (FoTBCA, EWY93729.1), Claviceps purpurea (CpTBCA, CCE31346.1), Colletotrichum graminicola (CgTBCA, EFQ26205.1), Co. orbiculare (CoTBCA, ENH78242.1), Verticillium dahliae (VdTBCA, EGY18367.1), Togninia minima (TmTBCA, XP_007910919.1), Neurospora crassa (NcTBCA, XP_964483.1), Magnaporthe oryzae (MoTBCA, XP_003709983.1), Gaeumannomyces graminis (GgTBCA, EJT76136.1), Marssonina brunnea (Mb510, XP_007289220.1), Blumeria graminis (BgTBCA, CCU76202.1), Neofusicoccum parvum (NpTBCA, XP_007583539.1), Aspergillus nidulans (AnTBCA, CBF70033.1), Candida albicans (CaTBCA, EEQ43562.1), Saccharomyces cerevisiae (ScRbl2, EGA72990.1), and Schizosaccharomyces pombe (SpAlp3, CAB40194.1). The phylogenetic tree was generated by the neighbor-joining method with 1000 bootstrap replicates using the Mega 4.1 software. B. Each TBCA orthologue contains one functional domain, tubulin binding cofactor A, which were identified by Pfam (http://pfam.xfam.org/). C. Alignment of the predicted amino acid sequences of TBCA domains from F. asiaticum, M. oryzae, N. crassa, A. nidulans, S. cerevisiae, S. pombe, and C. albicans. The Boxshade program was used to highlight identical (black shading) or similar (grey shading) amino acids. [file 12866_2015_374_MOESM4_ESM.tiff]

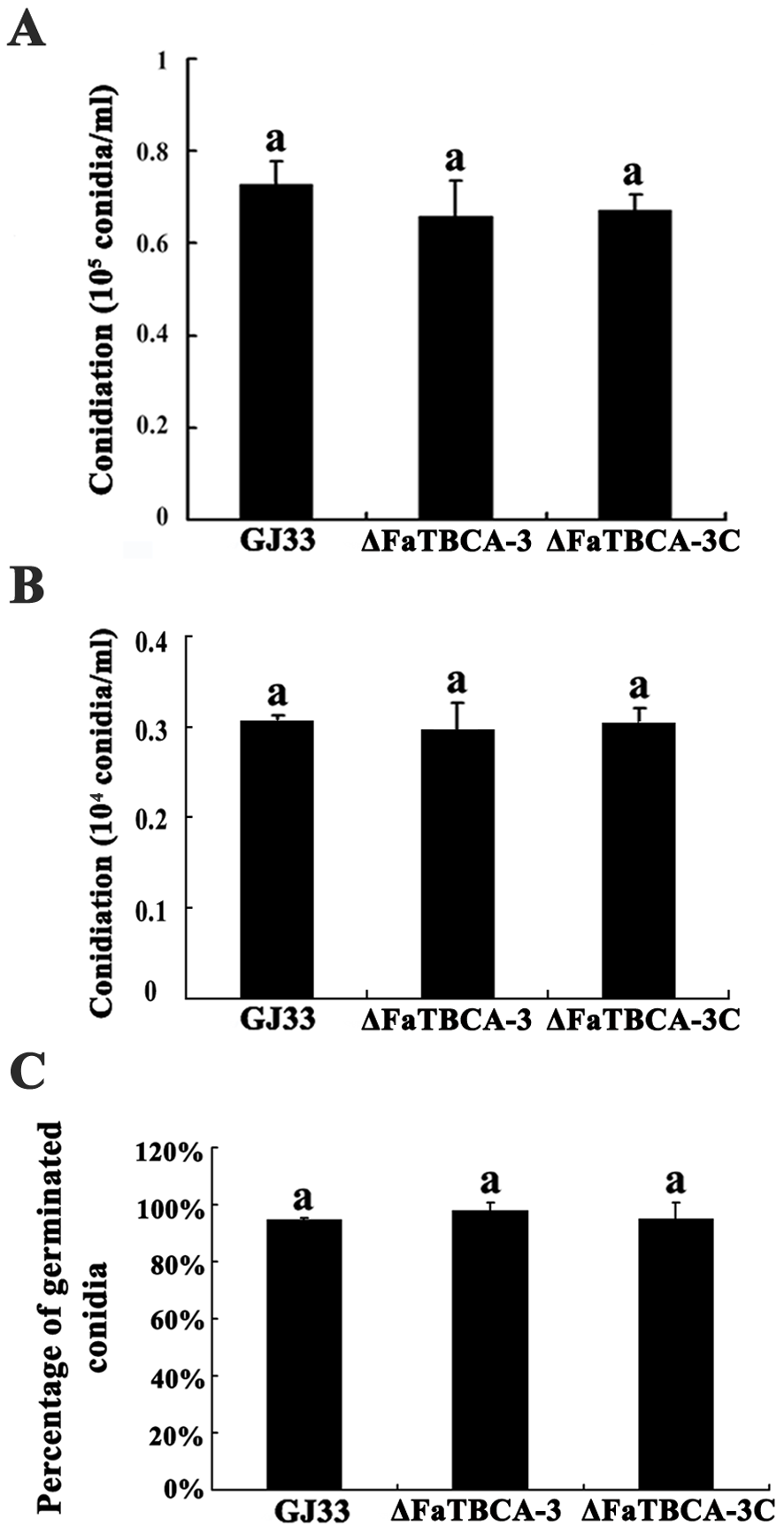

Supplement: Additional file 5: — Effect of FaTBCA deletion on conidiation and conidium germination of F. asiaticum. A. Conidia were counted after incubation of the wild-type GJ33, the mutant ΔFaTBCA-3 and the complemented strain ΔFaTBCA-3C in carboxymethyl cellulose liquid medium (CMC) for 5 days in a shaker at 25°C. B. Conidia were counted after incubation of each strain on mung bean agar (MBA) for one week of incubation at 25°C. C. Conidia of each strain were incubated in 2% (w/v) sucrose solutions and incubated at 25°C for 4 hrs. After incubation for 4 hrs, conidium germination of 150 conidia was examined. Line bars in each column denote standard errors of three repeated experiments. Bars with the same letter indicate no significant different according to a Fisher’s least significant difference (LSD) test at P = 0.05.T. [file 12866_2015_374_MOESM5_ESM.tiff]

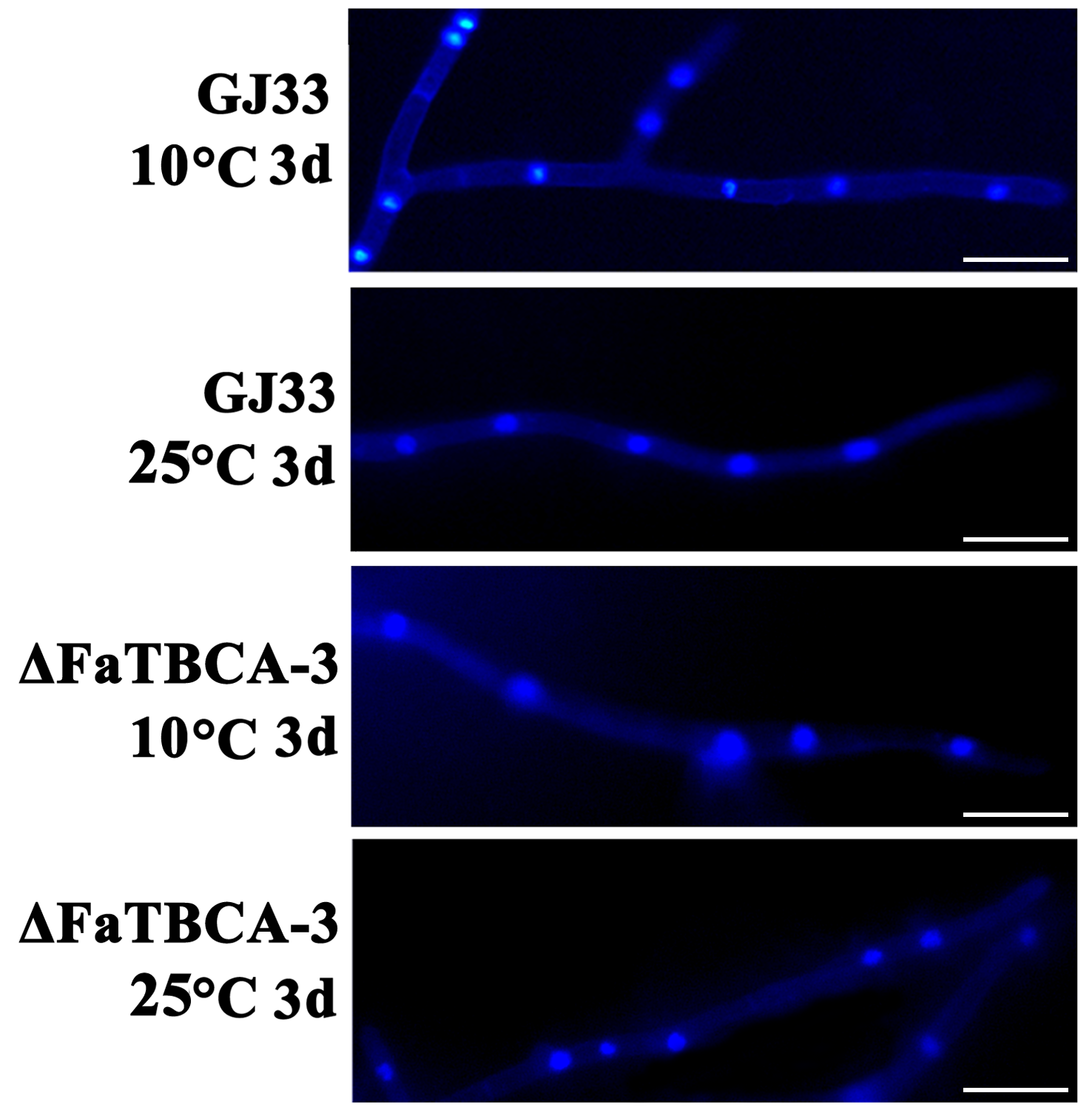

Supplement: Additional file 6: — The number of nuclei in ΔFaTBCA-3 does not change after incubation at 10 or 25°C. DIC images of nuclei in hyphal tip and middle section of the wild-type GJ33 and ΔFaTBCA-3 were taken after staining with 4',6-diamidino-2-phenylindole (DAPI). Bar = 10 μm. Each strain was incubated in PDB at 25°C for 1 day with a shaker then transferred to static incubation at 10 or 25°C for 3 days. [file 12866_2015_374_MOESM6_ESM.tiff]

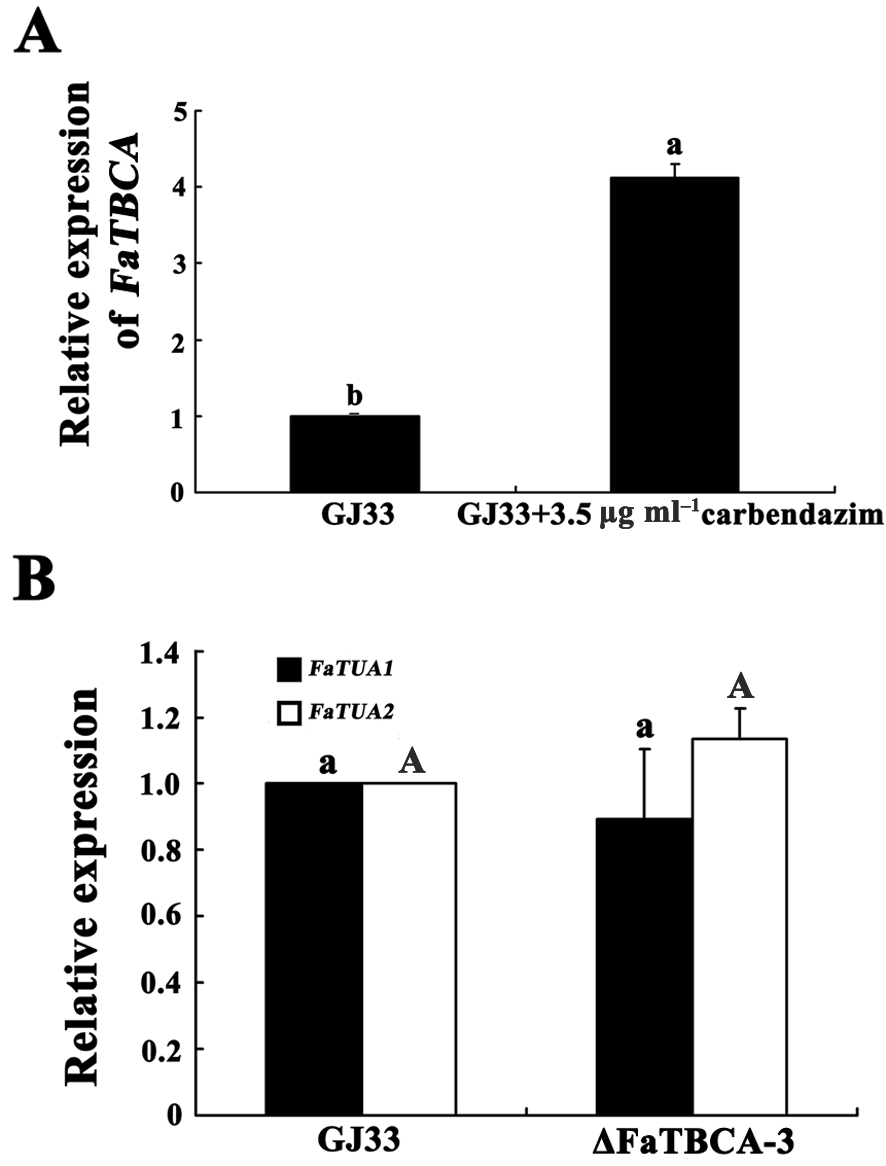

Supplement: Additional file 7: — The relative expression levels of FaTBCA treated with 3.5 μg ml −1 anti-microtubule drug carbendazim (A), FaTUA1 and FaTUA2 in the FaTBCA deletion mutant ΔFaTBCA-3 (B). Line bars in each column denote standard errors of three experiments. Values on the black bars followed by the same letter for each gene are not significantly different according to a Fisher’s least significant difference (LSD) test at P = 0.05. [file 12866_2015_374_MOESM7_ESM.tiff]
